# Taxonomy of Mitochondrial Cytochrome B Proteins of the Same Amino Acid Sequence Length

**DOI:** 10.1155/2021/1041818

**Published:** 2021-11-11

**Authors:** Alexander A. Zamyatnin, Tatiana A. Belozerskaya, Andrey A. Zamyatnin

**Affiliations:** ^1^A.N. Bach Institute of Biochemistry, Federal Research Center of Biotechnology, Russian Academy of Sciences, Moscow 119071, Russia; ^2^Institute of Molecular Medicine, Sechenov First Moscow State Medical University, Moscow, Russia; ^3^Belozersky Institute of Physico-Chemical Biology, Lomonosov Moscow State University, Moscow, Russia; ^4^Department of Biotechnology, Sirius University of Science and Technology, Sochi, Russia

## Abstract

Prior to this study, we discovered a protein characterized by many different amino acid sequences with the same number of amino acid residues. This turned out to be a unique cytochrome b, in which 1048 molecules out of 1689 contain 379 amino acid residues. A detailed study of the occurrence of this protein in living organisms at different taxonomic levels (from biological domains to biological orders of animals) has been carried out in the work presented here. We found that the main part of all b cytochromes is present in eukaryotes (99.2%), in biological kingdoms (95.9% in animals), in biological phylums (97.5% in chordates), and in biological classes (79.7% in mammals). Withal, this protein, containing 379 amino acid residues and characterized by many different amino acid sequences, is found only in eukaryotes (100%), only in animals (100%) and mainly in mammals (81.1%). Thus, a representative that has cytochrome b with a corresponding number of amino acid residues has not yet been identified among archaea and prokaryotes, while it is common in representatives of different biological types, classes, and orders of animals. It is believed that the structural diversity of a given protein within the same length and its one function of participation in the process of electron transfer relate to the physicochemical features of the extra- and intramembrane fragments of the polypeptide chain of this protein.

## 1. Introduction

The length (number of amino acid residues) of natural peptide structures varies over a very wide range. Thus, according to data from the protein-peptide database Swiss-Prot (https://www.uniprot.org/uniprot/?query=reviewed:yes), the minimum peptide structure consists of two, and the largest one consists of several tens of thousands of amino acid residues. Despite the fact that more than 500,000 natural amino acid sequences have already been described in detail (and despite their wide range of sizes), the length of protein molecules is rarely the subject of special consideration among other physicochemical characteristics.

Known investigations are mainly devoted to study of the shape of the distribution of peptide structures along the length within the natural range of the number of amino acid residues [1–4]. At the same time, various techniques are used to smooth out the irregularities of such distributions and to obtain the corresponding fitted mathematical expressions. Thus, peaks presented in the natural distribution are excluded from consideration, for which no explanation is given.

We have previously drawn attention to peaks in the distributions of different biological kingdoms and domains and have identified a unique protein which is represented by more than 1000 different amino acid sequences with a single length of 379 amino acid residues [5, 6]. This turned out to be mitochondrial cytochrome b, identified in numerous representatives of different biological species of animals. A detailed analysis of the occurrence of this protein in individual taxonomic groups of living organisms has been carried out in this work.

## 2. Methods

We used data from the Swiss-Prot protein-peptide database [7], which is a part of the UniProt database [8], in our investigation. The ability to perform different procedures and analyses is provided on the UniProt database website (https://www.uniprot.org/uniprot/?query=reviewed:yes). Thus, the UniProt database program tools were applied to our work highlighting all and specific amino acid residue sequences (option: Search), exclusion of sequence fragments (option: Sequence > Fragment > Sequence complete), extraction of specific sequences with a given number of amino acid residues p (option: Sequence > Sequence length > from p1 to p2), exclusion of identical sequences (option: Protein page > Similar proteins > 100% identity), and sorting protein names and sequences by different characteristics (standard UniProt table sorting).

At the time of the study, this contained information about 562,755 amino acid sequences, obtained for representatives of archaea, prokaryotes, and eukaryotes. The minimum number of amino acid residues (2) in it contains two oligopeptides [9, 10], and the maximum (35,213) contains one protein (mouse titin [11]). The Swiss-Prot database contains data not only on complete amino acid sequences but also on protein fragments. However, the database service makes it possible to exclude incomplete sequences. Data on 553,531 sequences were identified in the database after an appropriate procedure for the elimination of fragments. All b cytochromes known to date were isolated from these. The isolation and processing of data on these proteins has already been described in detail by us [6].

## 3. Results

**Figure 1:**
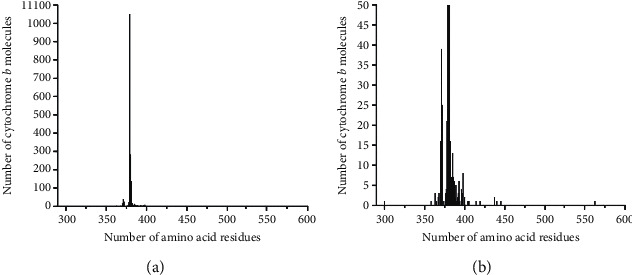
Distribution of the number of all identified cytochrome b molecules, according to data from the Swiss-Prot database on the number of amino acid residues. (a) Data at the ordinate, illustrating the entire maximum peak of the distribution. (b) The same data with a truncated ordinate and the same abscissa to identify all small peaks.

At the first stage, we identified all cytochrome b molecules of all living organisms from the entire dataset of the Swiss-Prot database. As a result, we were able to ascertain the distribution of 1689 of these cytochromes, according to the number of amino acid residues. It turned out that the number of amino acid residues contained in them can vary from 300 to 563. These data are illustrated in Figure 1(a), in which one large peak (corresponding to 1048 molecules containing 379 amino acid residues) stands out. In addition to this peak, 283 molecules made up of 380 residues, and 136 molecules made up of 381, can be identified. These are clearly visible in Figures 1(a) and 1(b). These figures also show a small number of molecules containing both more and fewer than 379 amino acid residues.

Let us consider these data in more detail in taxonomic groups at different levels. At the first (highest) level, we selected b cytochromes from the domains of archaea, prokaryotes, and eukaryotes [12]. Most known cytochrome b molecules were detected in eukaryotes (99.2%), as can be seen from the data in Table 1.

**Figure 2:**
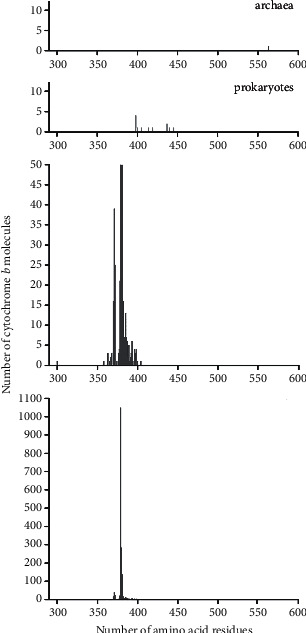
Distribution of the number of cytochrome b molecules, according to data from the Swiss-Prot database on the number of amino acid residues in various biological domains. The data for eukaryotes are presented at two scales of the ordinate—a truncated one, revealing small peaks throughout the entire region where lengths of amino acid residues exist, and a full one, where the entire maximum peak of the distribution is visible.

The same results are graphically presented in Figure 2. From the data in this figure and Table 1, it also follows that b cytochromes containing 379 amino acid residues are found only in eukaryotes (100%); the regions where these lengths occur in prokaryotes and eukaryotes overlap slightly, and the value of 563 amino acid residues for a single representative of archaea goes far beyond the values for both eukaryotes and prokaryotes. Thus, most of the known cytochrome b molecules are found in eukaryotes, and they contain 379 amino acid residues in most cases.

**Figure 3:**
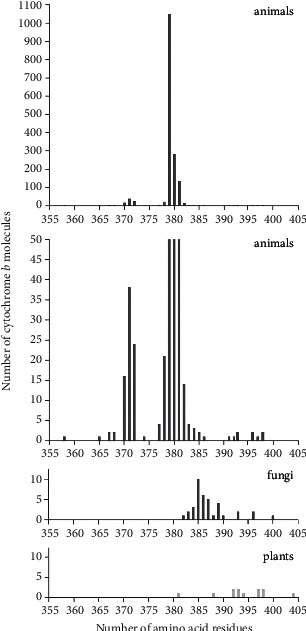
Distribution of the number of cytochrome b molecules, according to data from the Swiss-Prot database on the number of amino acid residues in various biological kingdoms. The data for animals are presented at two scales of the ordinate—a truncated one, revealing small peaks throughout the entire region where lengths of amino acid residues exist, and a full one, where the entire maximum peak of the distribution is visible.

Next, we analyzed data for 1676 b cytochromes of various biological kingdoms (only the eukaryotic domain). The results are shown in Table 2 in descending order according to the number of molecules detected. In this case, 95.9% of the molecules were identified in animals, and 65% (1048) of the molecules contained 379 amino acid residues.  Figure 3 shows how the intervals of the number of amino acid residues in animal fungi and plants relate to one another. It is clear that the regions where the lengths of amino acid residues occur in animals and fungi, as well as in animals and plants, do not overlap, while the overlap for plants and fungi is almost complete. The main result is that all b cytochromes of both plants and fungi contain more amino acid residues than the b cytochromes of animals. The size of the area where the lengths of cytochrome b occur in representatives of species not included in the considered kingdoms (300–391) turned out to be rather wide, overlapping with the area characteristic of animals and slightly overlapping with the same area in plants and fungi. In addition, none of them contained 379 amino acid residues; i.e., all proteins with this number of amino acid residues were found only in animal representatives.

**Figure 4:**
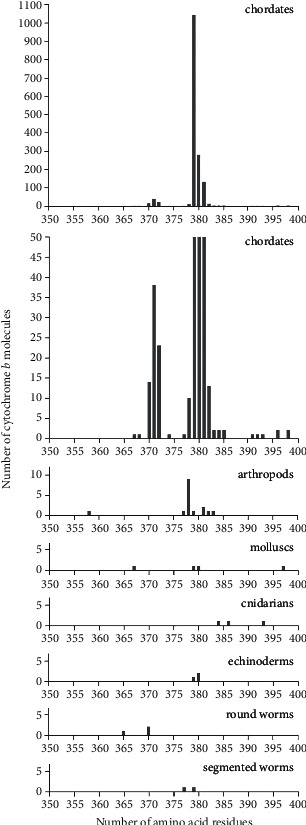
Distribution of the number of cytochrome b molecules, according to data from the Swiss-Prot database on the number of amino acid residues in various biological phyla. The data for chordates are presented at two scales of the ordinate—a truncated one, revealing small peaks throughout the entire region where lengths of amino acid residues exist, and a full one, where the entire maximum peak of the distribution is visible.

All b cytochromes of animals representing the biological phylum were considered at the next taxonomic level. In this case (Table 3 and Figure 4), it turned out that the overwhelming number of molecules of this protein is characteristic of representatives of the biological phylum of chordates (97.5%). In addition, it was found that the number of molecules containing 379 amino acid residues (1042) in chordates is 66%. At the same time, single molecules with this number of residues were also found in animals of other biological phyla (highlighted in bold in Table 3). These are represented by arthropods (migratory locust, *Locusta migratoria*), mollusks (spear squid, *Heterololigo bleekeri*), echinoderms (starfish, *Patiria pectinifera*), annelids (common earthworm, *Lumbricus terrestris*), and primary tracheal worms (velvet worm, *Epiperipatus biolleyi*).

**Figure 5:**
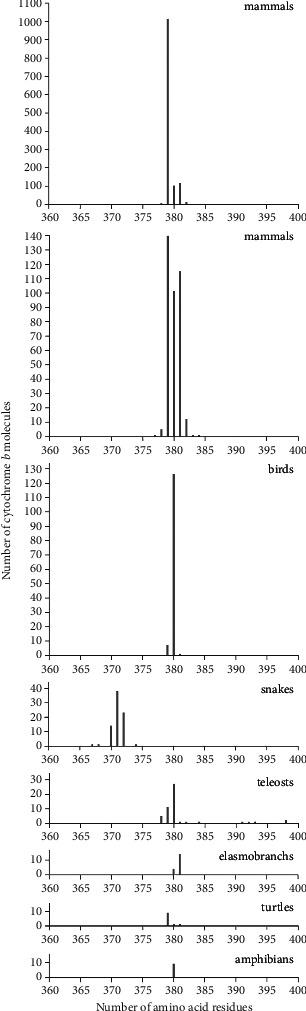
Distribution of the number of cytochrome b molecules, according to data from the Swiss-Prot database on the number of amino acid residues in various biological classes. The data for mammals are presented at two scales of the ordinate—a truncated one, revealing small peaks throughout the entire region where lengths of amino acid residues exist, and a full one, where the entire maximum peak of the distribution is visible.

We found b cytochromes to be the most abundant in mammals (79.7%) among biological classes of animals such as chordates, and the number of molecules containing 379 amino acid residues (1013) in mammals was 81.1% (Table 4 and Figure 5). The interval of the observed numbers of amino acid residues for these is less than 40, and the boundary values of this interval coincide with the number of residues in two lancelets: the common lancelet, *Branchiostoma lanceolatum*, and the Florida lancelet, *Branchiostoma florida*.

**Figure 6:**
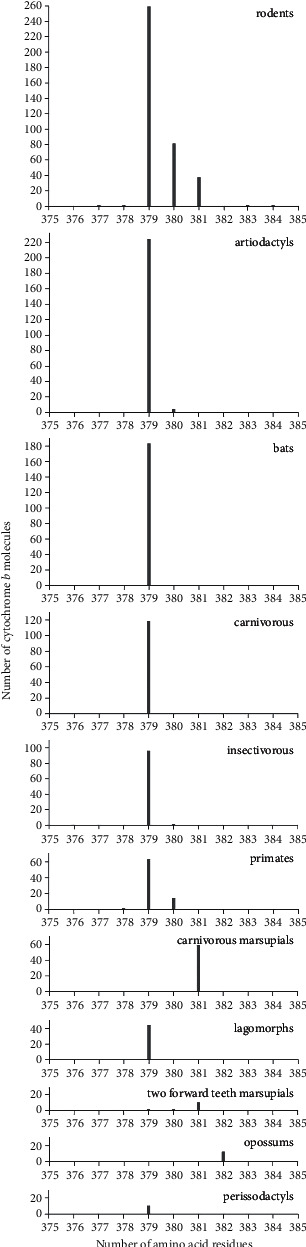
Distribution of the number of cytochrome b molecules, according to data from the Swiss-Prot database on the number of amino acid residues in various biological orders.

Analysis of the occurrence of b cytochromes containing 379 amino acid residues showed that most of them were found in representatives of rodents and artiodactyls (Table 5 and Figure 6). At the same time, representatives of bats, insectivores, and carnivores, as well as many orders represented by single molecules (Table 5), were always found to have only 379 residues.

## 4. Discussion

The data obtained indicate that mitochondrial cytochrome b, containing 379 amino acid residues and characterized by many different amino acid sequences, is found only in eukaryotes (100%), only in animals (100%) and mainly in mammals (79.7%). At the same time, the result of the analysis of different orders of mammals (Figure 6) does not allow us to assert that the representatives of one of the orders are characterized by the most frequent presence of this protein with this number of residues. According to the data given in Table 5, in most cases, the number of species with identified b cytochromes is less than the number of known species of this order. The only exceptions are the small orders *Peramelemorphia* (marsupial omnivores), *Scandentia* (tree shrews), and *Tubulidentata* (aardvarks). The ratio of the number of detected b cytochromes to the number of known biological species in this biological class (mammals) is also different in different orders. Therefore, the number of described b cytochromes may change significantly with further study of known, but not yet considered, mammals. Moreover, today, representatives of new biological species are constantly being discovered, including mammals, both fossils [16] and living mammals [17]. Therefore, we should expect continuing identification of more and more amino acid sequences of b cytochromes in newly discovered mammals and other living organisms. These data can change both the ratio of the number of all cytochrome b molecules in different biological orders and the ratio of molecules containing 379 amino acid residues.

However, the data currently available indicate that representatives of mammals have b cytochromes containing only 379 amino acid residues in half of all mammalian orders (Table 5, Figure 6), and the total number of such representatives is more than 80% (1013 out of 1249) of all animal b cytochromes. Note also that human cytochrome b is composed not of 379 residues, but of 380 [18]. At the same time, the number of residues is 379 [19] and 381 [20] in such widely studied animal species as the bovine and the mouse, respectively.

It is well known that peptide molecules of the same length, but different amino acid sequences, can have the same functions [21–24]. For example, the pentapeptides met-enkephalin YGGFM and leu-enkephalin YGGFL are natural ligands of opioid receptors [25]. Therefore, the existence of a large number of b cytochromes with 379 amino acid residues is apparently not surprising. However, the length of the protein alone cannot help us understand the special property that determines its effective functioning. It is known that the functional property of cytochrome b is its ability to participate in the process of electron transfer [26], and the structural feature (amino acid sequence) is its special properties that allow it to be a transmembrane protein [27]. Consequently, it is obvious that the functional property of cytochrome b depends not only on the length of the protein but also on the features of the structure of its extra- and intramembrane fragments. Therefore, a detailed study of the physicochemical features of the cytochrome b polypeptide chain along its entire length is required, along with analysis of the features of the primary structure.

## 5. Conclusions

In conclusion, it should be noted that the b cytochromes of representatives of biological species of archaea and prokaryotes were studied significantly fewer in number than representatives of eukaryotes (Table 1); representatives of plants and fungi were fewer in number than representatives of animals (Table 2); representatives of chordates were significantly greater in number than representatives of other biological types (Table 3); and there are more representatives of mammals than representatives of other biological classes (Table 4). Therefore, further investigation, adding to the knowledge gleaned by this study in terms of the b cytochromes of yet-to-be-studied biological species, may change the general understanding of the occurrence of this protein in different taxonomic groups. It can also be assumed that a coupled study of the biological and physicochemical characteristics of this protein will explain the reason for the anomalous value of 379 in terms of amino acid residues contained in most of the already known b cytochromes, while the relatively simple methodological approach described in this study can be useful for further search and identification of novel patterns of proteins similarities.

## Figures and Tables

**Table 1:** Content of the number of cytochrome b amino acid sequences in the swiss-prot database in various biological domains.

Biological domains	Number of molecules	Number of amino acid residues
*Archaea*	1	563
*Prokaryota* (bacteria)	12	398, 400, 405, 414, 419, 437, 440, 445
*Eukaryota*	1 676	300–404
All	**1 689**	**300–563**

**Table 2:** Content of the number of cytochrome *b* amino acid sequences in the Swiss-Prot database in various biological kingdoms of the eukaryote (*Eukaryota*) domain.

Biological kingdoms	Number of molecules	Number of amino acid residues
*Metazoa* (animals)	1 608	358–398
*Fungi* (fungi)	38	382, 383, 384, 385, 386, 387, 388, 389, 390, 400
*Viridiplantae* (plants)	12	381, 388, 392, 393, 394, 397, 398, 404
*Alveolata*	9	300, 363, 368, 376, 382, 391
*Euglenozoa*	3	363, 371, 372
*Amoebozoa*	3	385, 387, 389
*Rhodophyta*	2	381, 384
*Stramenopiles*	1	383
All *Eukaryota*	**1 676**	**300–404**

**Table 3:** Content of the number of cytochrome b amino acid sequences in the Swiss-Prot database in various biological phyla of animal (*Metazoa*) kingdom.

Biological phyla and other taxonomic groups of animals	Number of molecules	Number of amino acid residues
*Chordata* (chordate)	1 568	367–398
*Arthropoda* (arthropods)	16	358, 377, 378, **379**, 381, 382, 383
*Mollusca* (mollusks)	4	367, **379**, 380, 397
*Cnidaria* (cnidarians)	3	384, 386, 393
*Echinodermata* (echinoderms)	3	**379**, 380
*Nematoda* (nematodes)	3	365, 370
*Annelida* (annelids)	2	377, **379**
*Acanthocephalan* (thorny-headed worms)	1	372
*Bryozoan* (moss animals)	1	368
*Chaetognatha* (arrow worms)	1	378
*Entoprocta* (goblet worms)	1	378
*Onychophora* (velvet worms)	1	**379**
*Placozoa* (tricoplaxes)	1	383
*Priapulida* (penis worms)	1	377
*Rotifer* (rotifers)	1	**379**
*Xenacoelomorpha* (xenoturbellids)	1	380
All *Metazoa*	**1 608**	**358–398**

**Table 4:** Content of the number of cytochrome b amino acid sequences in the Swiss-Prot database in various taxonomic groups^a^ of chordate (*Chordata*) phylum.

Biological phyla and other taxonomic groups of animals	Number of molecules	Number of amino acid residues
*Mammalia* (mammals)	1 249	377–384
*Aves* (birds)	134	379–381
*Serpentes* (snakes)	78	367–374
*Teleostei* (teleosts)	51	378–398
*Elasmobranchii* (elasmobranchs)	18	380, 381
*Testudines* (turtles)	11	379–381
*Amphibia* (amphibians)	9	380
*Actinopterygii* (ray-finned fishes)	8	379, 380
*Cyclostomata* (jawless)	4	385, 396
*Lepidosauria* (reptiles)	2	379, 380
*Cephalochordata* (lancelets)	2	367, 398
*Archosauria* (reptiles)	1	383
*Coelacanthiformes* (lobefin fishes)	1	380
All *Chordata*	**1 568**	**367–398**

^a^Due to the incomparability of different data on the division of chordates into biological classes [13–15], we took arbitrary taxonomic groups used in the Swiss-Prot database.

**Table 5:** Content of the number of cytochrome b amino acid sequences in the Swiss-Prot database in various biological orders of mammal (*Mammalia*) order.

Biological orders of mammals	Number of molecules	Number of amino acid residues	Number of known species^a^
*Rodentia* (rodents)	381	377, 378, **379**, 380, 381, 383, 384	2 552
*Artiodactyla* (aetiodactyls)	228	**379**, 380	551
*Chiroptera* (bats)	183	**379**	1 386
*Carnivora* (carnivorous)	118	**379**	305
*Eulipotyphla* (insectivorous)	97	**379**, 380	527
*Primates* (primates)	78	378, **379**, 380	518
*Dasyuromorphia* (carnivorous marsupials)	59	381	78
*Lagomorpha* (lagomorphs)	44	**379**	98
*Diprotodontia* (two forward teeth marsupials)	12	**379**, 380, 381	155
*Didelphidae* (opossums)	12	382	111
*Perissodactyla* (odd-toed ungulates)	10	**379**	21
*Peramelemorphia* (marsupial omnivores)	5	381	5
*Caenolestidae* (rat opossum)	3	381	10
*Pilosa* (sloths and anteaters)	3	**379**	24
*Proboscidea* (elephants)	3	378	23
*Scandentia* (treeshrews)	3	**379**	3
*Monotremata* (monotremes)	2	**379**	7
*Afrosoricida* (tenrecs and golden moles)	1	**379**	55
*Cingulata* (armadillos)	1	**379**	20
*Dermoptera* (colugos)	1	**379**	2
*Hyracoidea* (hyraxes)	1	**379**	5
*Macroscelidea* (elephant shrews)	1	**379**	20
*Notoryctemorphia* (marsupial moles)	1	381	2
*Sirenia* (manatees and dugongs)	1	**379**	5
*Tubulidentata* (aardvarks)	1	**379**	1
*Microbiotheria* (monito del monte)	0	—	3
*Pholidota* (pangolins)	0	—	8
All *Mammalia*	**1 249**	**377–384**	**6 495**

^a^According to [14].

## Data Availability

Data can be made available on request.
